# Genome-wide identification and characterization of the galactinol synthase (*GmGolS*) gene family and its expression under drought, heat, and combined stress in soybean

**DOI:** 10.1186/s12870-026-09943-3

**Published:** 2026-10-07

**Authors:** Sobhi F. Lamlom, Nada S. Ibrahim, Nagy S. Radwan, Nader R. Abdelsalam

**Affiliations:** 1 Department of Plant Production, Faculty of Agriculture Saba Basha, Alexandria University, Alexandria, Egypt; 2 Agricultural Botany Department, Faculty of Agriculture, Alexandria University, Saba Basha, Alexandria, 21531 Egypt

**Keywords:** *GolS*, *Glycine max*, Genome-wide analysis, Phylogenetic analysis, Gene duplication, Stress tolerance

## Abstract

Galactinol synthase (GolS) catalyzes the first committed step in raffinose family oligosaccharide (RFO) biosynthesis and is implicated in plant adaptation to abiotic stress. Although GolS gene families have been characterized in several plant species, a comprehensive genome-wide analysis in soybean has been lacking. Here, we identified 25 *GmGolS* genes in the soybean genome, ranging from 328 to 644 amino acids in length, and characterized their genomic organization, evolutionary history, and stress-responsive expression. The 25 *GmGolS* genes were distributed unevenly across 11 chromosomes, with chromosomes 3 and 19 showing the highest gene density. Phylogenetic analysis with orthologs from *Arabidopsis thaliana* and *Oryza sativa* classified all GolS proteins into six groups, and structural conservation within groups suggests shared functional ancestry. Synteny analysis indicated that segmental duplication, supported by tandem clusters on chromosomes 4 and 5, appears to be the predominant mechanism underlying family expansion, with duplication events estimated to span from approximately 0.20 to 112.87 million years ago. Ka/Ks analysis of duplicate gene pairs indicated values predominantly below 1, consistent with purifying selection acting on this family since duplication. Subcellular localization predictions suggested diverse targeting to chloroplasts, the cytoplasm, and the plasma membrane, pointing to potential multi-compartment roles, while protein-protein interaction network analysis linked GmGolS proteins to carbohydrate-metabolism and stress-responsive partners, and homology modeling indicated a conserved GT8 fold consistent with the catalytic requirements for galactinol synthesis. To examine stress-responsive expression, RT-qPCR was performed on three soybean cultivars subjected to drought, heat, and combined stress at the seedling stage. *GmGolS* genes displayed variable and stress-specific expression patterns, with combined stress eliciting the strongest transcriptional response overall. *GmGolS-3*, *GmGolS-9*, *GmGolS-10*, *GmGolS-13*, and *GmGolS-17* showed comparatively higher expression across treatments, with *GmGolS-3* and *GmGolS-9* most strongly induced under combined stress; by contrast, *GmGolS-11* responded primarily to heat stress alone, and *GmGolS-13* was consistently downregulated across all treatments, suggesting functional divergence within the family. Taken together, these findings provide a foundation for understanding the evolution and stress-responsive functions of the *GmGolS* gene family in soybean and identify *GmGolS-3* and *GmGolS-9* as candidates warranting further investigation, though functional validation is required to confirm the precise biological roles of individual members.

**Supplementary Information:**

The online version contains supplementary material available at https://doi.org/10.1186/s12870-026-09943-3.

## Introduction

Global agricultural productivity is increasingly threatened by abiotic stresses, including drought, heat, salinity, and heavy metal toxicity, which collectively account for substantial crop yield losses worldwide [1, 2]. Climate projections indicate that the frequency and co-occurrence of these stressors will intensify over the coming decades, placing pressure on food systems and the capacity to sustain a growing global population [3–5]. In response, plants have evolved a range of physiological, biochemical, and molecular strategies to maintain cellular homeostasis and limit stress-induced damage [6]. Among these, the accumulation of low-molecular-weight compatible solutes, including amino acids, polyols, and soluble sugars, represents a conserved adaptive mechanism [7–9]. These solutes lower the osmotic potential of cells without disrupting normal metabolism, thereby protecting membrane integrity, stabilizing macromolecular structures, and scavenging reactive oxygen species (ROS) generated under stress [10].

Raffinose family oligosaccharides (RFOs), including galactinol, raffinose, stachyose, and verbascose, are among the more functionally diverse compatible solutes documented in plants [11]. RFOs serve multiple physiological roles: they function as primary carbon transport molecules in the phloem of certain species, protect embryos from desiccation during seed maturation, and accumulate in vegetative tissues in response to drought, cold, heat, and oxidative stress [11–16]. Beyond osmoprotection, RFOs appear to function as signaling molecules during pathogen attack and wounding, may contribute to systemic acquired resistance, and support carbohydrate catabolism during seed germination [17, 18]. Galactinol and raffinose also appear to protect cellular components from oxidative damage by scavenging ROS directly [19, 20]. Furthermore, RFO accumulation has been positively correlated with abscisic acid (ABA) biosynthesis, placing this metabolic pathway at the intersection of stress signaling and primary metabolism [21–23]. The rate-limiting step in RFO biosynthesis is catalyzed by galactinol synthase (GolS; EC 2.4.1.123), which transfers a galactosyl moiety from UDP-galactose to *myo*-inositol to yield galactinol, the primary galactosyl donor for subsequent RFO elongation reactions [24, 25]. GolS belongs to the glycosyltransferase family 8 (GT8) [24]. GolS-encoding genes have been identified in a range of plant species. In *Arabidopsis thaliana*, seven *AtGolS* genes have been functionally characterized: *AtGolS1* and *AtGolS2* are induced by drought and salinity, while *AtGolS3* responds specifically to cold stress [26]. Overexpression of *AtGolS2* in transgenic rice reportedly conferred improved drought tolerance and increased grain yield under field conditions [27]. In maize (*Zea mays*), three *ZmGolS* isoforms display distinct spatiotemporal expression patterns [28], and in rice (*Oryza sativa*), two GolS isoforms are regulated post-transcriptionally through stress-induced intron retention [27]. GolS genes have been identified in a wide range of other species, including cucumber [11], tomato [29], wheat [30], tea plant [31], sesame [32], cassava [33], tobacco [34], chickpea [35], citrus [36], and coffee [37], underscoring the conserved function of GolS-mediated galactinol accumulation in plant stress adaptation [38, 39].

Soybean (*Glycine max* (L.) Merr.) is a key agricultural crop worldwide, supplying plant protein and edible oil for both human use and animal feed [40–42]. It is cultivated on about 6% of the world’s farmland, making it one of the most extensively grown oilseeds [43, 44]. Nonetheless, soybean yields are vulnerable to abiotic stresses such as drought and heat, which impair cellular and metabolic functions during critical growth stages, leading to significant yield reductions [45, 46]. Despite its importance agriculturally, the molecular basis of stress adaptation in soybean remains incompletely characterized.

Although GolS genes have been studied in several plant species, no comprehensive genome-wide analysis of the GmGolS family in soybean has integrated gene family evolution, structural diversity, duplication history, conserved motifs, protein interaction networks, and stress-responsive expression under combined drought and heat stress. The potential role of *GmGolS* genes in coordinated responses to combined stresses is particularly underexplored. To address this gap, we applied an integrated bioinformatics and experimental approach to identify and characterize the *GmGolS* gene family in soybean. Specifically, we: (i) identified all 25 members of the *GmGolS* family using HMM profiling and BLASTP searches against the soybean reference genome; (ii) examined their chromosomal distribution, physicochemical properties, gene structure, conserved protein motifs, and predicted subcellular localization; (iii) inferred phylogenetic relationships and classified genes into subfamilies alongside orthologs from Arabidopsis thaliana, *Oryza sativa*, and *Zea mays*; (iv) evaluated cross-species collinearity to assess evolutionary conservation; (v) predicted protein-protein interaction networks; and (vi) examined the stress-responsive expression of selected *GmGolS* genes under drought, heat, and combined stress conditions in three soybean cultivars using RT-qPCR. These analyses provide a basis for understanding the structural diversity and potential functional roles of the *GmGolS* gene family, and may inform future breeding and biotechnological strategies targeting abiotic stress tolerance in soybean.

## Materials and methods

### Genome data retrieval and GolS gene identification

The complete genome sequence and annotation files (GFF3 format) for *Glycine max* (Williams 82, JGI Glycine max v4.1) were obtained from the Phytozome database v13.0 (https://phytozome-next.jgi.doe.gov/). We identified GolS family genes using a dual approach combining Hidden Markov Model (HMM) profiling and BLASTP sequence similarity searches. The *GmGolS* domain profile (PF00012) was retrieved from the Pfam database v35.0 (http://pfam.xfam.org/) and used for HMM-based searches with HMMER v3.3.2 integrated into TBtools v1.120 [47], applying an E-value cutoff of 1 × 10⁻⁵. In parallel, we performed BLASTP searches using known GolS protein sequences from Arabidopsis thaliana as queries against the soybean proteome, with an E-value threshold of 1 × 10⁻¹⁰, a word size of 6, and the BLOSUM62 substitution matrix. We merged and de-duplicated candidate sequences from both approaches. All identified proteins were subsequently validated using SMART v9.0 (http://smart.embl-heidelberg.de/) and InterProScan v5.59-91.0 (http://www.ebi.ac.uk/interpro/) to confirm the presence and structural integrity of conserved GolS domains. Only proteins containing complete domains, including both the nucleotide-binding and substrate-binding regions, were retained. We excluded sequences with premature stop codons or incomplete domain architecture, yielding a final curated set of 25 *GmGolS* genes.

### Physicochemical properties and subcellular localization prediction

Physicochemical properties of the 25 GmGolS proteins, including molecular weight (MW), theoretical isoelectric point (pI), grand average of hydropathicity (GRAVY), instability index, and aliphatic index, were computed using the ProtParam tool on the ExPASy server (https://web.expasy.org/protparam/). Proteins with an instability index below 40 were classified as stable. Subcellular localization was predicted using WoLF PSORT v0.2 (https://wolfpsort.hgc.jp/) with plant-specific parameters. Results were visualized as heatmaps in TBtools v1.120 using hierarchical clustering based on Euclidean distance and complete linkage. These predictions are computational in nature and require experimental validation before conclusions can be drawn about actual protein localization. This approach follows physicochemical- and localization-profiling methods previously applied to gene-family characterization in other crops [48].

### Chromosomal distribution and gene mapping

Chromosomal locations and gene coordinates were extracted from the soybean genome GFF3 annotation file and processed with TBtools v1.120. Physical positions of all *GmGolS* genes were mapped and visualized using the Gene Location Visualize function in TBtools. A comparable TBtools-based mapping workflow was used for genome-wide gene-family characterization in potato [49].

### Phylogenetic analysis and subfamily classification

The 25 *GmGolS* protein sequences were aligned with ClustalW v2.1 using default parameters (gap opening penalty = 10, gap extension penalty = 0.2, Gonnet substitution matrix). A phylogenetic tree was constructed using the Neighbor-Joining (NJ) method in MEGA X v10.2.6 with the Poisson correction model, pairwise deletion of gaps, and 1,000 bootstrap replicates to assess node support. The resulting tree was annotated and visualized using iTOL v6.7.4 (https://itol.embl.de/). Subfamily classification was based on phylogenetic clustering with bootstrap support values of 70% or higher; genes within well-supported clades were assigned to the same subfamily. This NJ/bootstrap-based classification approach follows that used for evolutionary analysis of transporter gene families in maize [50].

### Gene structure and conserved motif analysis

Exon–intron organization was visualized using the Gene Structure Display Server (GSDS) v2.0 (http://gsds.cbi.pku.edu.cn/), with coding sequences (CDS) and genomic sequences uploaded to generate structure diagrams illustrating the relative positions and sizes of exons, introns, and untranslated regions (UTRs). Conserved protein motifs were identified using the MEME Suite v5.5.0 (http://meme-suite.org/tools/meme) with the following parameters: maximum number of motifs = 10, minimum motif width = 6 amino acids, maximum motif width = 70 amino acids, and E-value threshold of 1 × 10⁻⁵. Identified motifs were functionally annotated against the NCBI Conserved Domain Database (CDD) and the Pfam database. A similar GSDS/MEME-based gene-structure and motif workflow was applied to a sugar-transporter gene family in passion fruit [51].

### Gene duplication analysis and evolutionary rate estimation

Gene duplication events were identified using MCScanX v1.0 within TBtools v1.120. Duplicated gene pairs were classified as tandem when located within 100 kb on the same chromosome, and as segmental when located on different chromosomes or separated by more than 100 kb on the same chromosome, following criteria widely used in plant genome duplication studies [52]. Synonymous (Ks) and non-synonymous (Ka) substitution rates for each duplicate pair were calculated using the Yn00 method in PAML v4.9, implemented through TBtools. The Ka/Ks ratio was used as an indicator of selective pressure, with values below 1 suggesting purifying selection, values near 1 consistent with neutral evolution, and values above 1 potentially reflecting relaxed constraint or positive selection. Divergence times were estimated from Ks using T = Ks/(2λ), with λ = 6.1 × 10⁻⁹ substitutions per synonymous site per year, a rate derived from EST-based comparisons of legume genomes, including soybean [53]. Pairs with Ks > 1.5 were flagged separately, since synonymous-site saturation reduces the precision of dating at high divergence, and are discussed as approximate lower-confidence estimates.

### Protein–protein interaction network analysis

Protein–protein interaction (PPI) networks for GmGolS proteins were constructed using the STRING database v12.0 (https://string-db.org/), with Glycine max as the target species. Both direct (physical) and indirect (functional) interactions were considered, applying a confidence score threshold of 0.4 (medium confidence). Network visualization and topological analysis were performed using Cytoscape v3.9.1. This STRING/Cytoscape-based PPI workflow follows that used for transporter gene families in maize [54].

### Three-dimensional protein structure prediction

Representative GmGolS protein structures were predicted by homology modelling using the SWISS-MODEL server (https://swissmodel.expasy.org/). Template selection was based on sequence identity and coverage. Model quality was assessed using the Global Model Quality Estimation (GMQE) score and the QMEAN composite scoring function. A comparable homology-modeling approach was used to characterize enzyme structures in barley [55].

### Plant materials and abiotic stress treatments

#### Plant materials and growth conditions

A controlled pot experiment was conducted at the Faculty of Agriculture, Saba Basha, Alexandria University, Egypt, during the summer growing season of 2025. Three soybean cultivars (Giza21, Giza 35 and Giza 111 ) were obtained from the Sakha Agricultural Research Station, Kafr El-Sheikh, Egypt. Seeds were surface-sterilized with 3% (v/v) sodium hypochlorite for 10 min, rinsed thoroughly with sterile distilled water, and placed on moistened filter paper for germination at 25 ± 2 °C. Transplanted pre-germinated seeds from each genotype into plastic trays (30 × 20 × 10 cm) filled with a sterilized mixture of field soil and farmyard manure (3:1, v/v). Seedlings were maintained in a controlled-environment growth chamber at 70% relative humidity and 24 ± 2 °C, under a 16-hour photoperiod with a photosynthetic photon flux density of 250–350 µmol m⁻² s⁻¹.

#### Abiotic stress treatments

Abiotic stress treatments were applied to 14-day-old soybean seedlings. For water-deficit (WD) stress, irrigation was withheld until soil moisture reached 15% of field capacity; leaf samples were collected at the onset of visible wilting symptoms, typically 3–5 days after treatment initiation. For heat stress (HS), seedlings were exposed to 42 °C in the growth chamber for durations of 4 h. Control plants were maintained at a constant 24 ± 2 °C throughout and were not subjected to the temperature changes used in stress treatments.

For combined stress (WD + HS), the chamber temperature was raised from 28 °C to 38 °C between 06:00 and 08:00, then lowered to 28 °C between 16:00 and 20:00. Drought conditions were maintained throughout the combined stress period by ceasing irrigation; no water was supplied during periods of heat exposure. The lower maximum temperature used in the combined treatment (38 °C, compared with 42 °C for HS alone) was intentional: simultaneous exposure to heat and drought imposes a greater physiological burden than heat alone, and a lower temperature ceiling was applied to prevent severe tissue damage and maintain plant viability during prolonged combined stress exposure. Control plants received normal irrigation with distilled water and were maintained at a constant 24 ± 2 °C.

#### RNA extraction and gene expression analysis

Leaf tissue (100 mg fresh weight) was harvested from stressed and control plants at defined time points and immediately snap-frozen in liquid nitrogen. Total RNA was extracted using TRIzol reagent (Invitrogen, USA) following the manufacturer’s protocol. RNA integrity and concentration were assessed using a NanoDrop 2000 spectrophotometer (Thermo Scientific, USA) and agarose gel electrophoresis. Only samples with A260/A280 ratios of 1.8-2.0 and distinct ribosomal RNA bands were used for downstream analysis. First-strand cDNA synthesis was performed from 2 µg of total RNA using the SuperScript III First-Strand Synthesis System (Invitrogen, USA). Gene-specific primers for all 25 *GmGolS* genes were designed using Primer3Plus software (http://www.primer3plus.com/) with the following criteria: primer length 18–25 bp; melting temperature 58–62 °C; GC content 40–60%; amplicon size 150–200 bp. We designed primers to target unique regions within the 3′ end of each gene’s coding sequence to maximize specificity. All primer pairs were validated against the *Glycine max* genome using NCBI Primer-BLAST to confirm single-target amplification. Prior to expression analysis, primer efficiency was verified by melting curve analysis (single peak), agarose gel electrophoresis (single band), standard curve analysis (efficiency 90–110%, R² > 0.98), and no-template controls. Supplementary Table S1 provides complete primer sequences, properties, and validation data.

Quantitative RT-qPCR was performed using SYBR Green PCR Master Mix (Applied Biosystems, USA) on an ABI 7500 Real-Time PCR System. Cycling conditions consisted of an initial denaturation at 95 °C for 10 min, followed by 40 cycles of 95 °C for 15 s and 60 °C for 1 min. Primer specificity was confirmed by post-amplification melting curve analysis. The soybean *GmActin11* gene (*Glyma.08G019700*) was used as the internal reference for normalization. All RT-qPCR experiments were performed with three independent biological replicates, each prepared by pooling leaf tissue from at least 20 plants to minimize biological variability. Each biological replicate was analyzed in three technical replicates to ensure reproducibility. Relative expression levels were calculated using the 2⁻^ΔΔCt^ method [56] with fold changes expressed relative to the control condition. Genes were considered differentially expressed when both statistical significance (*P* < 0.05, one-way ANOVA followed by Tukey’s HSD test) and a fold-change threshold (|log₂(fold change)| ≥ 1) were satisfied simultaneously; genes meeting only one criterion were not classified as differentially expressed. Statistical analyses were performed in R v4.2.0.

### Statistical analysis and data visualization

All statistical analyses were conducted using R software v4.2.0. Pearson’s correlation coefficient was used for correlation analyses. Heatmaps were generated using the pheatmap package with hierarchical clustering. Statistical significance was defined as *P* < 0.05 for all tests. Figures and visualizations were produced using TBtools v1.120, R v4.2.0 with the ggplot2 package, and Adobe Illustrator 2023. All color schemes were designed to be colorblind-accessible and suitable for publication.

## Results

### Identification and characterization of *GmGolS* gene family members

A total of 25 GolS genes were identified in the soybean genome using the BLASTP program, with known *AtGolS* amino acid sequences as references. Simultaneously, hidden Markov model (HMM) analyses were performed. Candidate genes identified by these methods were confirmed using multiple domain databases, including PFAM, SMART, and NCBI’s Conserved Domain Database (CDD). All candidates possessed complete *GmGolS* domains, verifying their membership in the *GmGolS* family. They were systematically numbered from *GmGolS-1* to *GmGolS-25* based on their chromosomal positions. Characterization of their physical and chemical properties showed notable diversity: protein lengths ranged from 328 (*Glyma.10G145300*) to 644 amino acids (*Glyma.02G238200*), with theoretical molecular weights between 37.57 and 74.96 kDa. The isoelectric points (pI) varied from 5.24 to 9.22, indicating diverse charge properties within the family. The GRAVY scores ranged from − 0.448 to 0.29, mostly negative or near-neutral, consistent with hydrophilic properties. Stability analysis indicated that all 25 proteins are stable (instability index < 40), suggesting structural stability across various cellular conditions. The aliphatic index ranged from 76.45 to 104.09 (average 88.674), reflecting differences in thermostability. Detailed data for each family member are available in Supplementary Table S2.

### Chromosomal localization and genomic distribution

**Fig. 1:**
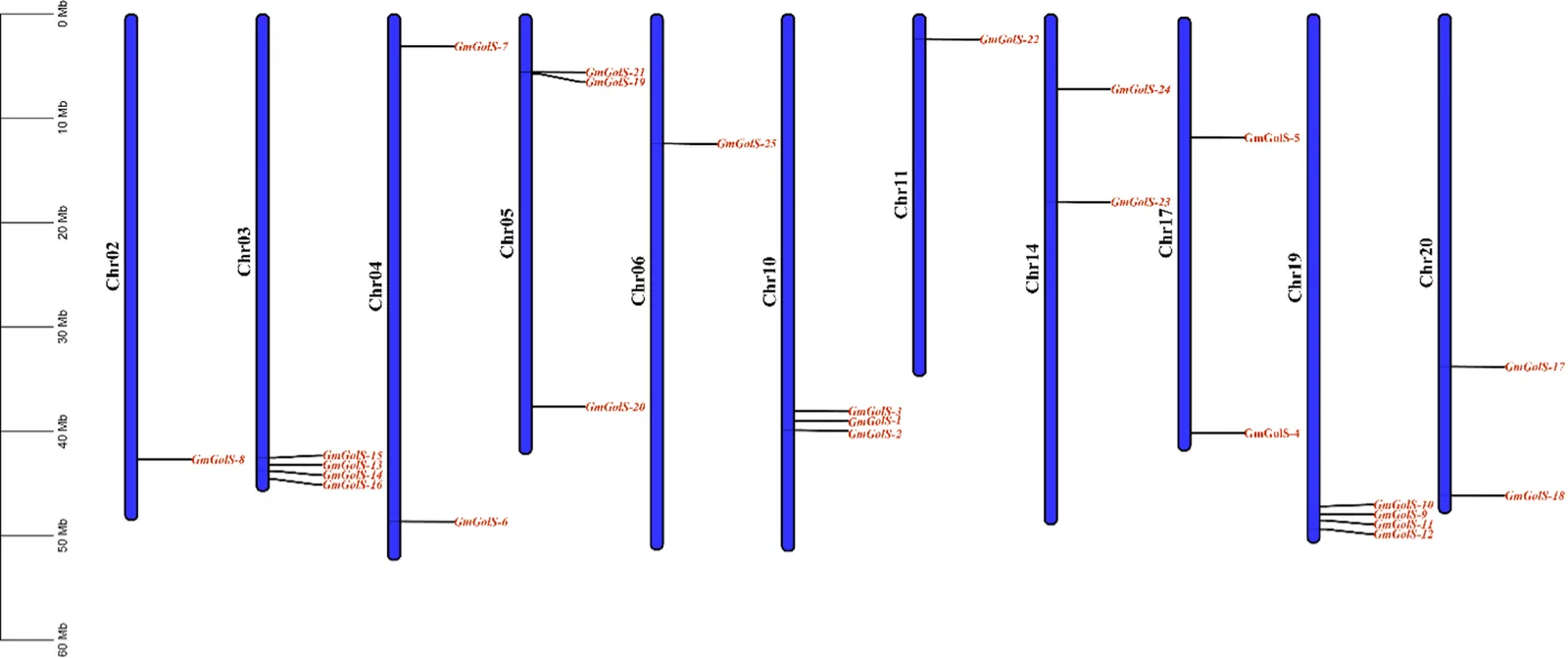
Chromosomal locations of *GmGolS* genes in the soybean genome. The physical map displays 25 *GmGolS* genes distributed across 11 soybean chromosomes (*GmGolS-1* to *GmGolS-25*). Chromosomes are depicted as vertical bars scaled to their physical lengths (Mb). The uneven distribution suggests a role for segmental and tandem duplication in expanding this gene family

To examine the genomic organization and distribution of *GmGolS* genes in the soybean genome, chromosomal localization was analyzed using genomic coordinates from the soybean reference genome (Fig. 1). The genome-wide analysis identified 25 GolS genes scattered unevenly across 11 soybean chromosomes. This irregular distribution suggests that *GmGolS* genes may have undergone selective retention or loss on specific chromosomes during soybean genome evolution. The distribution varied significantly, with Chr03 and Chr19 containing the highest number of GolS genes. Chromosomes 5 and 10 each host three GolS genes, while chromosomes Chr04, Chr14, Chr17, and Chr20 each contain two GolS genes. In contrast, chromosomes Chr02, 6, and 11 each contain only one gene.

### Phylogenetic relationships and subfamily classification

**Fig. 2:**
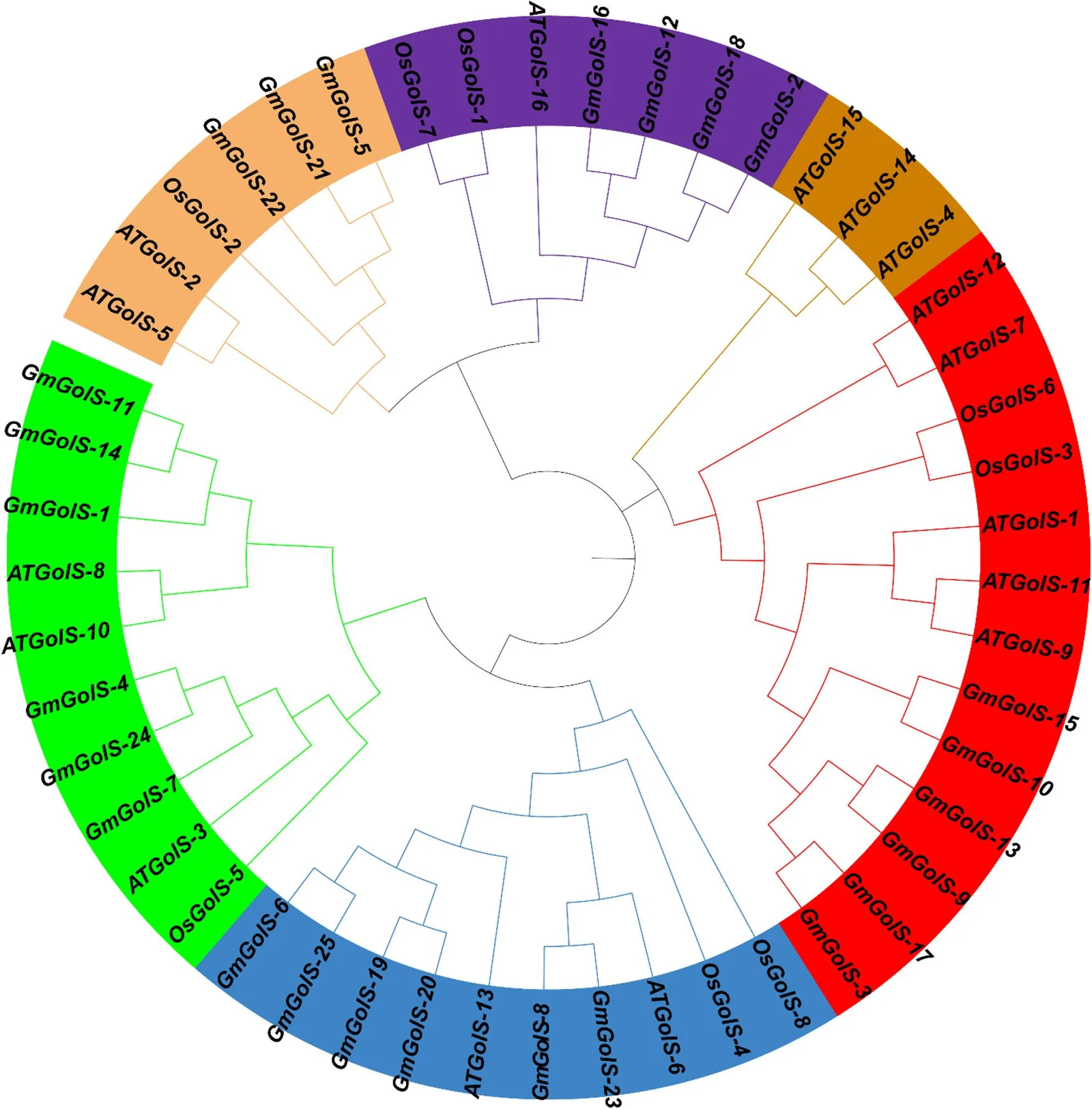
Phylogenetic relationship of GolS genes in Soybean, *Arabidopsis thaliana*, and *Oryza sativa*. The tree was constructed using the neighbor-joining method in MEGA X with 1,000 bootstrap replicates. All GolS members are divided into six groups, each represented by a distinct color (purple, brown, red, blue, green, and yellow)

A neighbor-joining (NJ) phylogenetic tree was constructed from an alignment of 25 *GmGolS*, 16 *AtGolS* (*Arabidopsis thaliana*), and 8 *OsGolS* (*Oryza sativa*) protein sequences, with 1,000 bootstrap replicates (Fig. 2). The 49 GolS proteins examined were assigned to six groups (purple, brown, red, blue, green, and yellow) based on phylogenetic clustering with bootstrap support values of 70% or higher. Genes within each group generally shared similar structures, and groups differed by conserved domain composition and exon-intron organization. The red, blue, and green groups each contained six *GmGolS* members and were among the largest by total gene count. The purple group comprised four *GmGolS* genes, while the yellow group, with three *GmGolS* members, was the smallest. The brown group contained only *Oryza sativa* GolS genes, suggesting greater evolutionary divergence from the soybean and Arabidopsis members. The phylogenetic groupings largely match previous multi-species GolS analyses and suggest that functional specialization may accompany the structural divergence observed across subfamilies.

### GmGolS protein domain and motif analysis

**Fig. 3:**
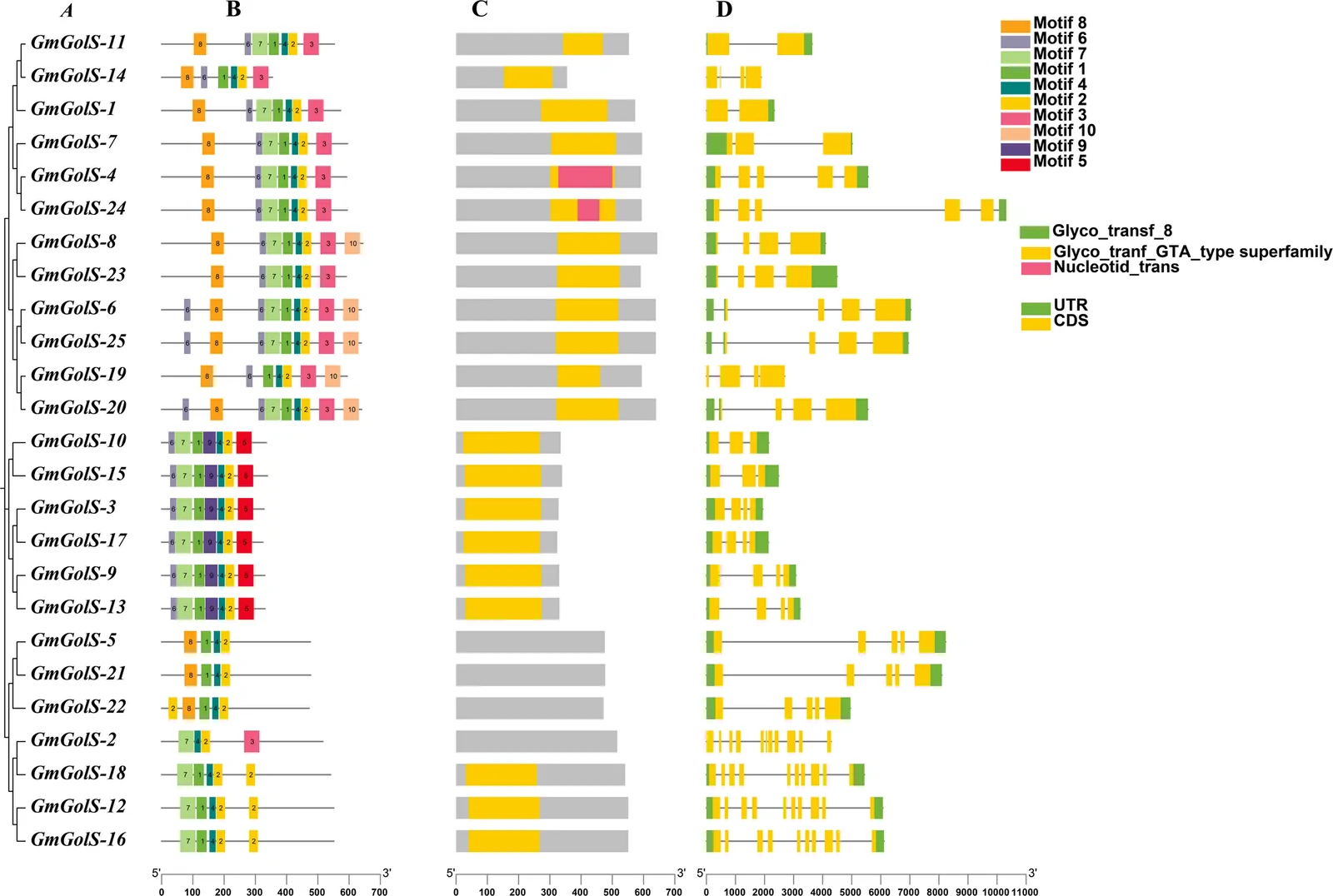
GmGolS protein motif organization and domain architecture. (**A**) Schematic representation of motif distribution across GmGolS protein sequences (scale: 0-700 amino acids). Ten conserved motifs (Motif 1–10) are shown in the 5′ to 3′ orientation. (**B**) Detailed view of motif arrangement in the N-terminal region. (**C**) Complete GmGolS protein architecture (scale: 0–10,000 nucleotides), showing the relationship among conserved motifs, the GolS superfamily domain, UTRs, and CDS. (**D**) Summary of motif conservation patterns across the 25 GmGolS sequences

Detailed analysis of gene structure, domain architecture, and conserved motifs were performed for all 25 *GmGolS* genes (Figs. 3 A-D). Gene lengths ranged from compact structures of under 700 bp to more complex arrangements of approximately 10,000 bp. Members within the same clade showed consistent motif composition, suggesting functional conservation within subfamilies. The presence of Glyco_transf_8 (PF00535) and Glyco_transf_GTA_type superfamily domains in all candidates confirms their identity as galactinol synthases, consistent with their role in RFO biosynthesis. Gene structure analysis revealed considerable diversity in intron-exon arrangements. Some gene clusters ( *GmGolS-10* to *GmGolS-13*) are relatively compact with few introns, whereas others (, *GmGolS-24*) exhibit more extensive intronic regions, possibly reflecting transposable element insertions or genomic rearrangements following duplication. The presence of UTRs in some genes may indicate post-transcriptional regulatory complexity. The structural conservation observed within subfamilies, alongside diversity between them, is consistent with a history of whole-genome duplication (WGD) followed by sub-functionalization within the *GmGolS* family. Subcellular localization was predicted for 25 GmGolS proteins using WoLF PSORT v0.2 with plant-specific settings (Figure S1; Table S3).

### Chromosomal collinearity and cross-species synteny analysis

**Fig. 4:**
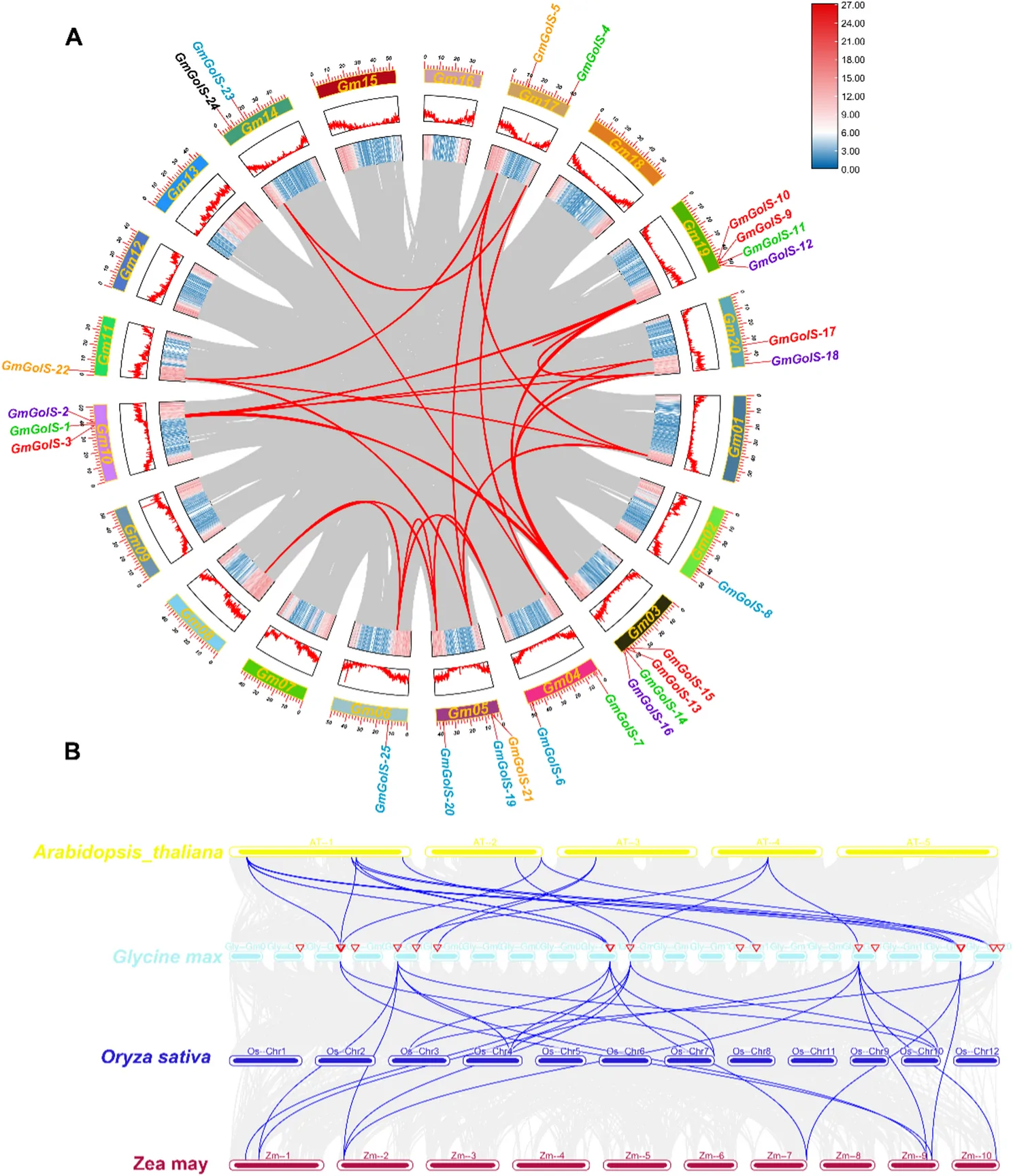
Chromosomal collinearity and cross-species synteny of *GmGolS* genes. (**A**) Circos plot of intra-genomic collinearity across the soybean genome. Gray ribbons represent all genome-wide syntenic blocks; red ribbons connect collinear *GmGolS* gene pairs. (**B**) Cross-species collinearity plot showing syntenic relationships among four plant genomes. Red arrowheads indicate GmGolS positions on the *Glycine max* track

Intra-genomic collinearity analysis and cross-species synteny analysis were performed to examine the mechanisms underlying the expansion of the *GmGolS* gene family and to assess the evolutionary conservation of GolS genes across plant lineages. The circos plot (Fig. 4A) provides a genome-wide view of syntenic relationships among the 25 *GmGolS* genes. Red connecting lines highlight collinear *GmGolS* gene pairs, most of which span non-homologous chromosomes, suggesting segmental duplication as the predominant mechanism underlying the expansion of the *GmGolS* family. Dense connectivity is observed among genes on chromosomes Gm02, Gm03, Gm05, Gm17, Gm19, and Gm20, consistent with the gene clustering observed on chromosomes 3 and 19. Long-range connections linking Gm02/Gm03-resident genes (*GmGolS-1*,* -2*,* -3*) with Gm19-resident genes (*GmGolS-9*,* -10*,* -11*,* 12*) implicate the two WGD events known to have shaped the soybean genome. Tandem gene clusters are also apparent, particularly on chromosomes Gm04 and Gm05, indicating that tandem duplication has contributed to secondary expansion. Cross-species collinearity analysis among *Arabidopsis thaliana*, *Glycine max*, *Oryza sativa*, and *Zea mays* (Fig. 4B) revealed extensive collinear connections between *Arabidopsis* and *Glycine max*, with most *GmGolS* loci traceable to syntenic regions in *Arabidopsis*, reflecting shared dicot ancestry and conservation of GolS function since divergence. Collinear relationships with *O. sativa* are also evident, though less dense, consistent with the greater evolutionary distance between these lineages (150 Mya). Connections between *O. sativa* and *Z. mays* show a distinct pattern compared with dicot comparisons, reflecting the independent evolutionary trajectories of monocot genomes. Collectively, these patterns suggest that the core GolS function is broadly conserved across angiosperms, while the expansion to 25 members in soybean appears to be a lineage-specific outcome of successive polyploidization and gene retention.

### Ka/Ks analysis and estimation of duplication times

Ka/Ks ratios were calculated for duplicated *GmGolS* gene pairs to assess the selective pressures acting on this family (Table S4). The overwhelming majority of pairs showed Ka/Ks values below 1, consistent with purifying selection and functional conservation following duplication; only one pair (*Glyma.02G238200.1–Glyma.14G122600.1*) showed Ka/Ks above 1, which we interpret with caution given the small sample underlying that estimate rather than as confirmed positive selection.

Estimated duplication times fell into three groups. A subset of pairs (Ks < 0.5, e.g., *Glyma.19G247000.1–Glyma.03G249500.1* at 7.15 Mya) date to within the timeframe of the known recent soybean whole-genome duplication (13 Mya). A second, smaller group (Ks 0.5–1.0) corresponds to the older soybean/legume duplication event (59 Mya). The majority of pairs, however, show Ks values above 1.0, yielding estimated times from roughly 80 to over 160 Mya, predating soybean’s own duplication history. Given the deep conservation of the GolS gene family across plant lineages, these older pairs most plausibly represent paralogs retained from the eudicot-wide γ whole-genome triplication event rather than a soybean-specific duplication. Estimates in this range, particularly those with Ks above 1.5–2, should nonetheless be treated as approximate given the reduced precision of Ks-based dating at high sequence divergence.

### Protein–protein interaction network and structural analysis

**Fig. 5:**
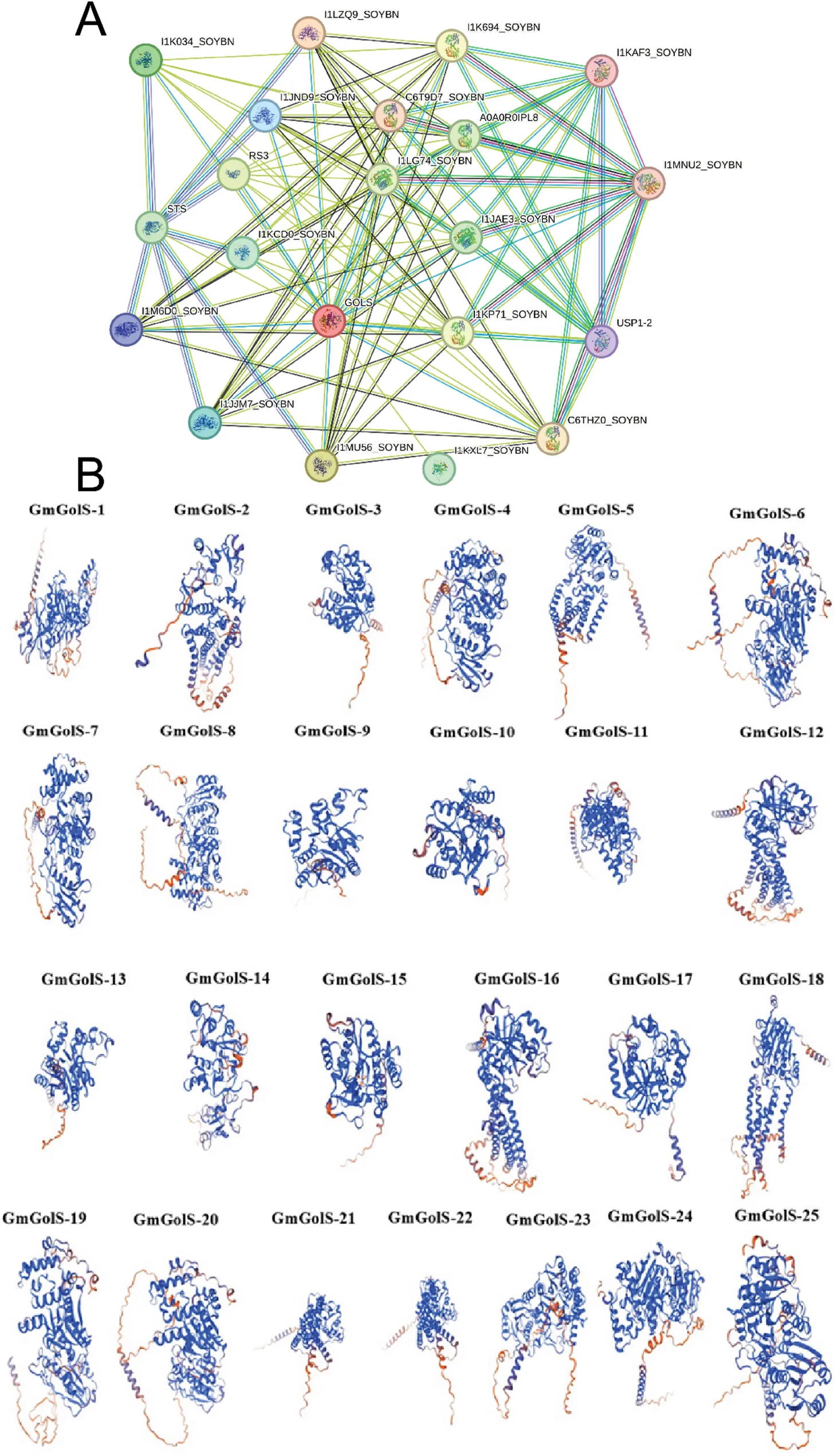
Protein-protein interaction network and structural analysis of soybean GmGolS proteins. (**A**) PPI network generated using the STRING database, showing GmGolS proteins and their predicted interaction partners. Line thickness indicates interaction confidence. (**B**) Three-dimensional homology model of a representative GmGolS protein, showing the characteristic GT8 fold with alpha-helical core (blue) and flexible loop regions (orange)

PPI network analysis using the STRING database revealed that GmGolS proteins are embedded in functional networks involving carbohydrate metabolism and stress-responsive pathways (Fig. 5A). Key interaction partners include RS3, STS, and USP1-2, as well as several predicted soybean proteins (I1KCD0_SOYBN and I1JAES3_SOYBN). These interactions align with the known role of GolS in RFO biosynthesis and suggest potential links between this pathway and broader metabolic and stress-signaling networks. GolS also appears to occupy a regulatory position linking RFO biosynthesis to inositol metabolism. Homology modeling of representative GmGolS proteins (Fig. 5B) revealed the characteristic GT8 fold, with a stable core of alpha-helices and flexible loop regions. This architecture matches the catalytic requirements for galactinol synthesis and supports substrate binding and RFO production. These structural observations complement the functional annotation provided by the PPI network and support the potential relevance of *GmGolS* genes in stress tolerance contexts.

### Expression profiling of *GmGolS* genes under abiotic stress

**Fig. 6:**
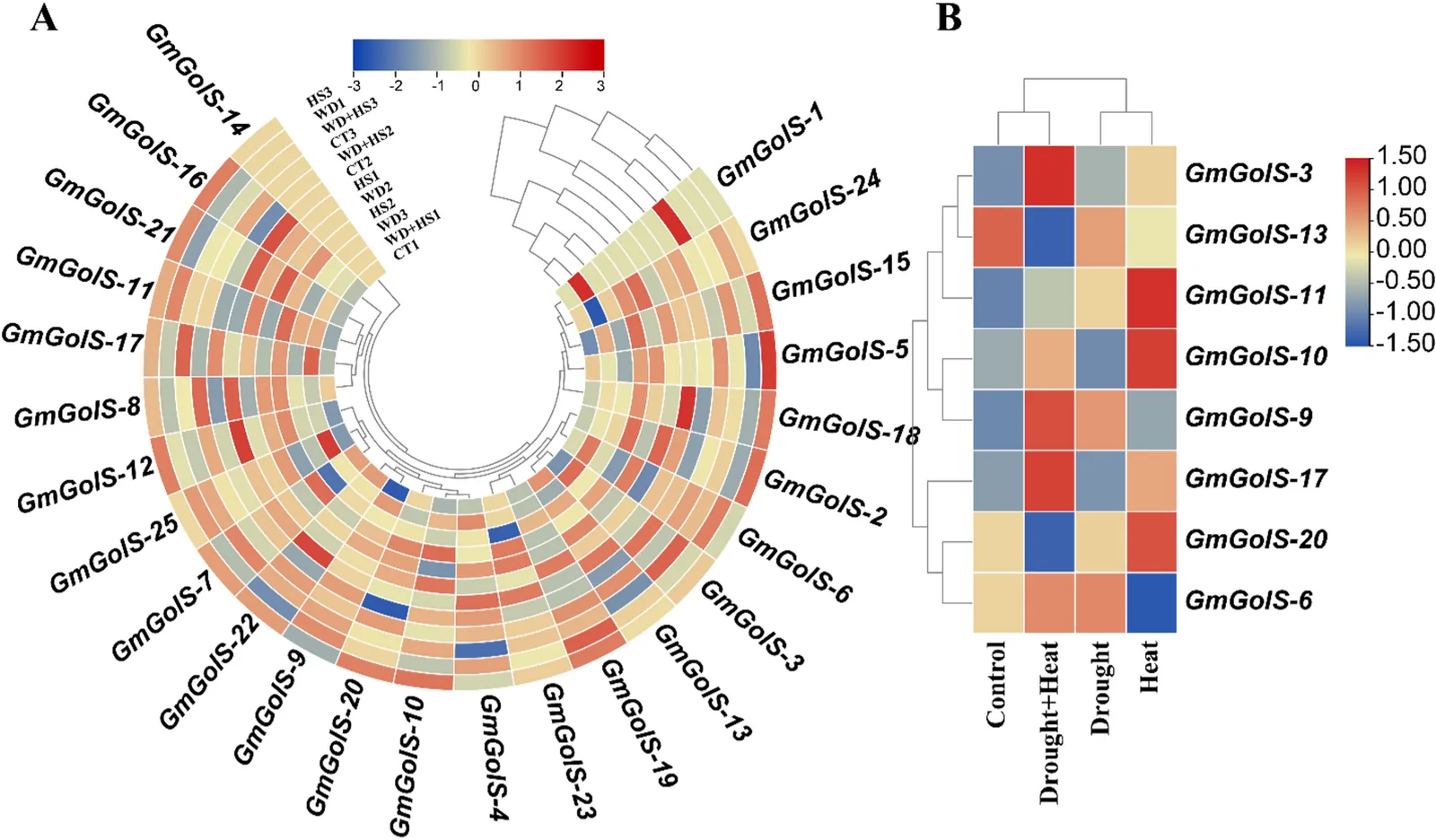
Expression profiles of *GmGolS* genes under heat (HS), drought (WD), and combined (WD + HS) stress conditions. (**A**) Circular heatmap showing global transcriptional patterns of 25 *GmGolS* genes. Color scale ranges from low (blue) to high (red) normalized expression. (**B**) Hierarchically clustered heatmap of the most responsive genes, divided into groups based on expression similarity

Expression profiles of the 25 *GmGolS* genes were examined under drought (WD), heat (HS), and combined stress (WD + HS) using heatmap visualization and quantitative RT-qPCR (Figs. 6A-B). The global heatmap (Fig. 6A) revealed varied expression patterns across stress conditions, suggesting functional diversity within the gene family. A substantial proportion of *GmGolS* genes were upregulated under stress treatments, particularly under combined stress (WD + HS), while a subset showed stable or reduced expression. Quantitative analysis identified several genes with higher expression levels across treatments, including *GmGolS-3*, *GmGolS-10*, *GmGolS-13*, and *GmGolS-17*. Among these, *GmGolS-3* and *GmGolS-9* showed elevated transcript levels under combined stress (WD + HS), suggesting that their transcriptional activation may be associated with coordinated responses to simultaneous abiotic stressors. By contrast, *GmGolS-11* showed elevated expression under heat stress alone but was reduced under combined stress, indicating stress-specific regulatory patterns. *GmGolS-20* expression was less consistent across conditions, suggesting a more context-dependent regulatory role.

The clustered heatmap of the most responsive genes (Fig. 6B) grouped *GmGolS-3* and *GmGolS-9* in a cluster characterized by marked induction under combined stress. *GmGolS-11* responded primarily to individual stresses. Genes including *GmGolS-10*, *GmGolS-17*, *GmGolS-6*, and *GmGolS-20* showed moderate or variable expression. *GmGolS-13* was consistently downregulated across all treatment conditions, suggesting a negative regulatory role or functional divergence within the family. Taken together, these patterns suggest that combined stress elicits a stronger transcriptional response than individual stresses, and that *GmGolS-3* and *GmGolS-9* may warrant further investigation as candidates in soybean abiotic stress tolerance programs, pending functional validation.

## Discussion

### Gene family size and structural conservation

The identification of 25 *GmGolS* genes in soybean is consistent with the expanded gene family sizes observed in other polyploid species. For comparison, the GolS family sizes include seven members in *Arabidopsis*, nine in poplar [57], 20 in *B. napus*, and nine in *N. tabacum* [34]. The variation in gene numbers across species likely reflects differences in genome size and evolutionary history. Several *GmGolS* genes displayed complex intron-exon architectures, and the diversity in gene structure between subfamilies may reflect distinct regulatory strategies. Structural conservation within subfamilies, however, suggests that functional constraints have been maintained during evolution, consistent with shared ancestry and potentially overlapping roles in RFO biosynthesis or stress responses [58, 59].

### Chromosomal distribution and evolutionary history

The uneven distribution of *GmGolS* genes across chromosomes, with pronounced clustering on chromosomes 3 and 19, is consistent with tandem and segmental duplication as primary drivers of gene family expansion. Ka/Ks analysis indicated that most duplicate pairs have likely evolved under purifying selection, implying that functional redundancy following duplication has been reduced through sub-functionalization or neo-functionalization rather than wholesale gene loss. A small number of pairs with Ka/Ks ratios near or above 1 may reflect episodes of relaxed selection, though additional statistical validation would be needed to characterize these cases more precisely. The wide range of estimated duplication times, from ancient events exceeding 100 Mya to much more recent duplications, suggests that *GmGolS* family expansion has been an ongoing process throughout soybean evolution [60].

### Phylogenetic relationships and functional implications

The classification of GolS proteins into six major groups, supported by bootstrap values of 70% or higher, is broadly consistent with prior analyses across angiosperm taxa. Within each group, and conserved exon-intron organization support the phylogenetic classification and suggest that structural constraints, likely reflecting functional requirements, have been maintained during diversification. The presence of *GmGolS* members in groups containing Arabidopsis and rice orthologs implies conservation of core GolS function across species, while group-specific expansions in soybean may reflect lineage-specific adaptations. The absence of soybean members from the brown group, which contains only Oryza sativa sequences, indicates greater divergence between monocot and dicot GolS clades.

### Predicted subcellular localization and functional diversity

The predicted subcellular localization patterns suggest that GmGolS proteins may operate across multiple cellular compartments, including the cytoplasm, chloroplast, and plasma membrane. This diversity in predicted targeting is consistent with the multifunctional roles proposed for GolS proteins in RFO biosynthesis and stress responses across different cellular contexts [61]. However, these are computational predictions generated by WoLF PSORT and have not been experimentally confirmed; interpret the localization data with caution and treat them as hypotheses requiring further investigation [62, 63]. The compartment-specific distribution patterns observed in the heatmap clustering may reflect evolutionary specialization, but empirical validation using fluorescent tagging or subcellular fractionation assays is needed before drawing firm conclusions.

### Protein-protein interactions and cellular networks

The PPI network centered on GmGolS proteins suggests that these enzymes may be integrated into broader metabolic and stress-signaling networks beyond their primary role in RFO biosynthesis. Predicted interactions with RS3, STS, USP1-2, and several uncharacterized soybean proteins (I1KCD0_SOYBN, I1JAES3_SOYBN) are consistent with the hypothesis that GolS occupies a regulatory node connecting RFO biosynthesis, inositol metabolism, and carbohydrate-mediated stress responses [11, 36]. The RFO accumulation function, including protection of embryos from dehydration and signaling roles during biotic stress [18, 38, 39, 64], is well-supported in the literature, and the interaction data presented here provide computational support for similar roles in soybean, though direct experimental verification is needed.

### Stress-responsive expression and candidate genes

The observed expression patterns support a potential role for *GmGolS* genes in adaptation to abiotic stress and are broadly consistent with the known functions of GolS genes in other plant species. The upregulation of multiple *GmGolS* members under combined stress (WD + HS) is consistent with the hypothesis that RFO biosynthesis may contribute to membrane protection, osmotic adjustment, and ROS scavenging under stress conditions [26, 65]. Among the genes examined, *GmGolS-3* and *GmGolS-9* showed the most pronounced transcriptional activation under combined stress, making them promising candidates for further functional investigation. Their phylogenetic clustering with stress-responsive GolS members from soybean, maize, and other species [26, 37, 66, 67], together with the presence of ABA- and abiotic stress-responsive cis-elements in their predicted promoter regions, provides some support for a functional role in stress adaptation. The similarity in expression patterns between these two genes and other known stress-responsive GolS members, along with evidence of partial functional conservation in duplicated pairs, further supports this interpretation.

It is important, however, to note that these conclusions are primarily based on transcriptional profiling, which is not sufficient on its own to establish direct causal roles in stress tolerance. Transcriptional changes may reflect downstream consequences of stress signaling rather than primary regulatory functions. The consistent downregulation of *GmGolS-13* across all stress conditions is notable and may indicate functional divergence within the family, possibly reflecting tissue-specific or condition-dependent roles observed for certain GolS isoforms in previous studies [12, 68]. The greater transcriptional responses observed under combined stress relative to individual stresses align with the concept of unique combined-stress transcriptional signatures, which frequently diverge from responses to individual stressors in plants.

Taken together, the integration of phylogenetic, structural, and expression data provides a useful overview of the potential functional landscape of the *GmGolS* gene family in soybean [26, 69]. However, to confirm the biological roles of candidate genes, including *GmGolS-3* and *GmGolS-9*, additional experimental approaches are required, such as gene overexpression, VIGS, CRISPR/Cas-mediated mutagenesis, or hairy root transformation assays under controlled stress conditions.

### Study limitations

Several limitations of the present study should be acknowledged. First, we inferred the functional roles of candidate genes primarily from in silico analysis and transcript expression profiling; we did not experimentally validate gene function. Second, subcellular localization and protein interaction networks are based on computational predictions and have not been verified experimentally. Third, we conducted RT-qPCR validation under controlled laboratory conditions, which may not fully capture the complexity of field environments. Finally, identifying *GmGolS-3* and *GmGolS-9* as priority candidates rests solely on expression data; further validation using genetic and biochemical approaches is needed to confirm their roles. These limitations notwithstanding, the findings establish a useful foundation for targeted functional studies and applied breeding efforts in soybean.

## Conclusions

A genome-wide characterization of the galactinol synthase (GolS) gene family in soybean (*Glycine max*) identified 25 *GmGolS* genes distributed unevenly across 11 chromosomes. Bioinformatic analysis confirmed the presence of conserved Glyco_transf_8 (PF00535) and Glyco_transf_GTA_type superfamily domains in all members, indicating structural conservation consistent with shared GolS function. Phylogenetic and duplication analyses suggest that purifying selection has shaped the *GmGolS* family, with evidence of ongoing diversification through both ancient WGD events and more recent gene duplications. Predicted subcellular localization and PPI data, treated as supporting bioinformatic evidence rather than experimentally validated results, are consistent with involvement in stress-related pathways and carbohydrate metabolism. Expression profiling by RT-qPCR revealed that *GmGolS* genes display stress-specific and variable expression patterns, with combined stress (WD + HS) generally eliciting stronger transcriptional responses than individual stressors. Among the genes examined, *GmGolS-3* and *GmGolS-9* showed the highest transcriptional activation under combined stress, suggesting they may be involved in coordinated abiotic stress responses in soybean. These candidates merit further functional investigation, though confirmatory experiments, including gene overexpression, VIGS, and CRISPR-based approaches, will be required to establish their precise biological roles. This study provides a useful reference for the *GmGolS* gene family and identifies several genes as starting points for future functional characterization. The data may also inform marker-assisted breeding strategies to improve soybean resilience to abiotic stresses that increasingly threaten crop productivity under changing climatic conditions.

## Supplementary Information

Supplementary Material 1.

Supplementary Material 2.

## Data Availability

All sequence data used in this study are publicly available from the Phytozome database (https://phytozome-next.jgi.doe.gov/). The complete dataset, including gene sequences, annotation files, and analysis results are available as supplementary materials. Raw data and analysis scripts are available from the corresponding author upon reasonable request.
