# Supplementary material for: Genome-wide identification and characterization of the galactinol synthase (GmGolS) gene family and its expression under drought, heat, and combined stress in soybean
Source: BMC Plant Biol. 2026 Oct 7;26:1751. doi: 10.1186/s12870-026-09943-3 (PMC13660067; doi:10.1186/s12870-026-09943-3)
Supplement: Supplementary file 1 [file 12870_2026_9943_MOESM2_ESM.docx]

**Predicted Subcellular Localization**

Subcellular localization was predicted for 25 GmGolS proteins using WoLF PSORT v0.2 with plant-specific settings (Figure 3; Table S3). Eleven cellular compartments were assessed, including the nucleus, cytoplasm, mitochondria, chloroplasts, endoplasmic reticulum, plasma membrane, Golgi apparatus, peroxisomes, vacuoles, cytoskeleton, and extracellular space. Predicted localization patterns varied among proteins, with the majority showing higher scores in the cytoplasm, chloroplast, and plasma membrane. Proteins with comparable predicted localization profiles were grouped using hierarchical clustering based on Euclidean distance and complete-linkage. These results represent computational predictions and should be interpreted with appropriate caution, as they have not been experimentally validated. Rather than a major experimental finding, this analysis provides supporting bioinformatic evidence to contextualize the functional diversity of the *GmGolS* family across cellular compartments.

**Figure S1. Predicted subcellular localization of 25 *GmGolS* proteins**. The heatmap shows WoLF PSORT prediction scores across 11 compartments (Nucl, nucleus; Cyto, cytoplasm; Mito, mitochondria; Vacu, vacuole; Cysk, cytoskeleton; Chlo, chloroplast; E.R., endoplasmic reticulum; Plas, plasma membrane; Golg, Golgi apparatus; Pero, peroxisome; Extra, extracellular). Scores range from 0 to 10; higher values indicate greater predicted confidence for a given compartment. Gray cells indicate low or baseline prediction scores. Hierarchical clustering used Euclidean distance and complete linkage. We generated all predictions with WoLF PSORT v0.2 using plant-specific parameters and visualized them in TBtools v1.120.
